# Hypertrophy Dependent Doubling of L-Cells in Roux-en-Y Gastric Bypass Operated Rats

**DOI:** 10.1371/journal.pone.0065696

**Published:** 2013-06-11

**Authors:** Carl Frederik Hansen, Marco Bueter, Nadine Theis, Thomas Lutz, Sarah Paulsen, Louise S. Dalbøge, Niels Vrang, Jacob Jelsing

**Affiliations:** 1 Department of Histology, Gubra, Hørsholm, Denmark; 2 Department of Human Nutrition, University of Copenhagen, Frederiksberg, Denmark; 3 Department of Visceral and Transplant Surgery, University Hospital Zurich, Zurich, Switzerland; 4 Center of Integrative Human Physiology, University of Zurich, Zurich, Switzerland; 5 Institute of Veterinary Physiology, Vetsuisse Faculty, University of Zurich, Zurich, Switzerland; INRA, France

## Abstract

**Background and Aims:**

Roux-en-Y gastric bypass (RYGB) leads to a rapid remission of type 2 diabetes mellitus (T2DM), but the underlying mode of action remains incompletely understood. L-cell derived gut hormones such as glucagon-like peptide-1 (GLP-1) and peptide YY (PYY) are thought to play a central role in the anti-diabetic effects of RYGB; therefore, an improved understanding of intestinal endocrine L-cell adaptability is considered pivotal.

**Methods:**

The full rostrocaudal extension of the gut was analyzed in rats after RYGB and in sham-operated controls ad libitum fed or food restricted to match the body weight of RYGB rats. Total number of L-cells, as well as regional numbers, densities and mucosa volumes were quantified using stereological methods. Preproglucagon and PYY mRNA transcripts were quantified by qPCR to reflect the total and relative hormone production capacity of the L-cells.

**Results:**

RYGB surgery induced hypertrophy of the gut mucosa in the food exposed regions of the small intestine coupled with a doubling in the total number of L-cells. No changes in L-cell density were observed in any region regardless of surgery or food restriction. The total gene expression capacity of the entire gut revealed a near 200% increase in both PYY and preproglucagon mRNA levels in RYGB rats associated with both increased L-cell number as well as region-specific increased transcription per cell.

**Conclusions:**

Collectively, these findings indicate that RYGB in rats is associated with gut hypertrophy, an increase in L-cell number, but not density, and increased PYY and preproglucagon gene expression. This could explain the enhanced gut hormone dynamics seen after RYGB.

## Introduction

The prevalence of severe obesity is rapidly increasing and has reached epidemic proportions [1]. It is associated with significant co-morbidities including type 2 diabetes mellitus (T2DM), hyperlipidemia, arterial hypertension, coronary artery disease, and liver dysfunction [2]. Bariatric surgery, and in particular Roux-en-Y gastric bypass (RYGB), is currently the most widely used intervention method to achieve sustained weight loss in morbidly obese subjects resulting in a substantial improvement in co-morbidities and cardiovascular outcome [3], [4]. A remarkable feature of RYGB is the immediate improvement of T2DM even before significant body weight loss occurs [5], [6], [7], [8]. Thus, body weight loss per se cannot explain the marked anti-diabetic effect following RYGB. Although the exact mechanisms underlying the profound anti-diabetic effects of RYGB remain to be determined, it is generally assumed that gut hormones such as glucagon-like-peptide 1 (GLP-1) and peptide YY (PYY) play an important role [9], [10]. Both hormones are produced and secreted primarily in the intestinal tract by endocrine L-cells and previous studies have consistently shown increased plasma levels of GLP-1 and PYY following RYGB, lending support for the involvement of these hormones in the immediate metabolic response to RYGB [11], [12]. In an attempt to separate the effect of a substantial weight loss from the direct effect of surgery, Laferrère et al showed that RYGB surgery increased the “incretin effect” in humans about 5-fold compared to unoperated body-weight matched controls [13]. Data also suggest that the early increase in insulin release after RYGB is mediated by other mechanisms than just body weight loss [14].

RYGB surgery involves a significant rearrangement of the small intestine, but the post-operative effect on intestinal morphology is not fully understood. Several types of intestinal-surgery can lead to profound hormonal and morphological changes. Accordingly, it has long been recognized that gut resection or inflammation can lead to both morphological and hormonal changes in the gut [15], [16]. Intestinal resection is associated with increased circulating levels of enteroglucagon and glicentin in rats [17], [18]. In agreement, also ileal, but not colonic, resection in humans leads to increase in enteroglucagon [15]. Furthermore, epithelial proliferation has been also observed in both rats [19] and humans [20] after biliopancreatic bypass surgery, a type of bariatric surgery in which parts of the small bowel are bypassed.

A few studies have examined the density of endocrine cells after various types of bariatric surgery. Jejunoileal bypass surgery in obese humans has been coupled to a selective increase in the density of CCK and somatostatin cells in the duodenum [21]. In addition, Mumphrey and coworkers [22] recently demonstrated an increased thickness of the gut mucosa coupled with unchanged density of CCK, serotonin and GLP-1 positive cell profiles following RYGB in diet induced obesity (DIO) rats, thus providing an indirect evidence for an adaptive increase in the number of endocrine cells. Significant mucosal hypertrophy of the small bowel after bariatric surgery in rats has previously been demonstrated by Bueter et al [23]. Furthermore, intestinal hypertrophy has also been found after biliopancreatic diversion in rats [24]. Such morphological and cellular alterations may be linked to changes in the number and function of enteroendocrine cells leading to increased plasma hormone levels after RYGB. Hence, improved understanding of the morphological changes following bariatric surgery is considered important to understand and possibly further improve these types of surgery.

To test the hypothesis that an altered gut hormonal profile after RYGB is associated with an increased enteroendocrine cell number, we used our established RYGB rat model [23], [25], [26] and unbiased stereological methods to in-depths investigate the quantitative morphological changes in the gut after RYGB, as well as alterations in the number and distribution of endocrine L-cells in the intestine.

## Materials and Methods

### Animals

Fifteen male normoglycemic Wistar rats (Harlan Laboratories Inc., Blackthorn, UK; Elevage Janvier, Le-Genest-St. Isle, France) weighing 432±7.1 g were individually housed under a 12 h/12 h light-dark cycle at a room temperature of 21±2°C. Water and standard chow was available ad libitum, unless otherwise stated. Animal experiments were performed at the University of Zürich and were approved by the Veterinary Office of the Canton Zurich, Switzerland.

Rats were given one week of acclimatization before being assigned to RYGB (n = 5) or sham-operation (n = 10). After surgery, rats received liquid diet for 3 days before access to normal chow was reintroduced. The sham-operated group was then randomly divided into two groups. One was fed ad libitum (SHAM), the other was food restricted to maintain body weight similar to the RYGB rats (SHAM WM). Food intake was measured manually at least twice per week throughout the duration of the study. Based on experiences from previous studies [23], SHAM WM rats were given approximately 15 g of chow diet daily, which was offered at dark onset and readjusted every third day depending on the body weight development of the SHAM WM compared to the RYGB rats.

### Surgery

**Figure 1:**
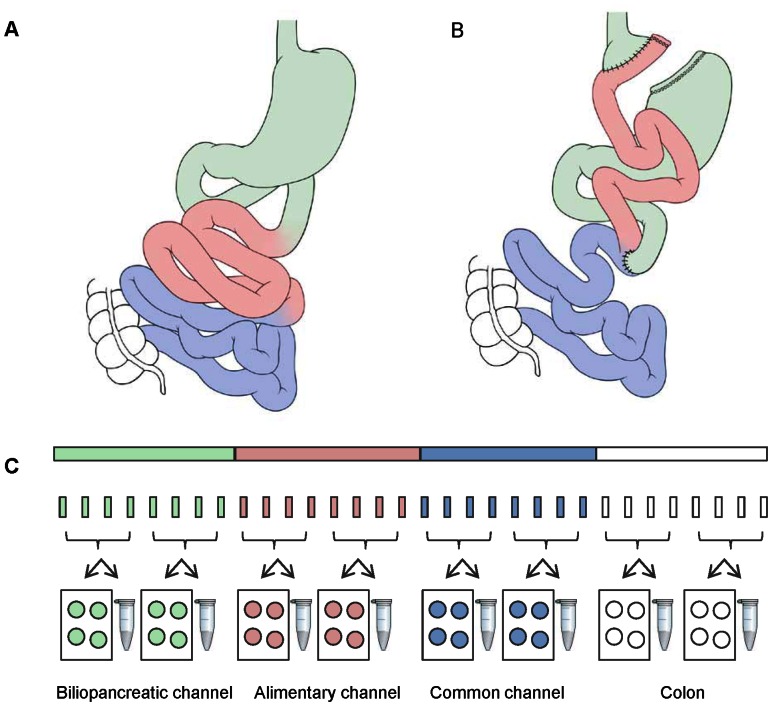
RYGB surgery and sampling procedure. The RYGB surgical procedures connecting the upper jejunum (alimentary channel, red) to the gastric pouch circumpassing the duodenum (biliopancreatic channel, green) (A–B). At termination each segment was sampled using stereological sampling principles into two sets of 7–9 transverse biopsies. One set was used for histology, one set for qPCR analyses (C).

Surgery was performed according to an established protocol as previously described [27]. Briefly, rats were food deprived overnight while water was available ad libitum. Prior to surgery, rats were weighed, and then anesthetized with isofluorane (4% for induction, 3% for maintenance). Preoperatively, 0.4 ml/kg Enrofloxacin and 1 mg/kg Flunixin were administered intraperitoneally (ip) as prophylaxis for postoperative infection and pain relief, respectively. In the sham-operated group, a 7 mm gastrotomy on the anterior wall of the stomach and a 7 mm jejunotomy were performed with subsequent closure. In the RYGB group, the proximal jejunum was divided 15 cm distal to the pylorus to create a biliopancreatic channel. After identification of the caecum, the ileum was then followed proximally to create a common channel of 25 cm. Here, a 7 mm side-to-side jejuno-jejunostomy between the biliopancreatic channel and the common channel was performed (Figure 1 A–B).

### Sampling, Embedding and Sectioning

The entire small bowel including duodenum and complete colon was sampled on the day of study termination (23 weeks after surgery) to ensure similar categorization of the respective segments. The biliopancreatic channel, the alimentary channel, the common channel as well as the complete colon were carefully separated in RYGB operated rats and frozen rapidly on dry ice. Corresponding segments of jejunum, duodenum, and ileum of sham-operated rats were identified and separated by the same surgeon who performed the surgery. Each of the four regions was subsequently sampled into two sets of transverse biopsies (∼4 mm of length) while still frozen (**Figure 1** **C**). The sampling of biopsies was carried out using systematic uniform random sampling principles ensuring an unbiased representation of the entire region (7–9 samples in total) in each sample set [28]. One set was kept frozen and processed for RNA gene expression analysis. The other set of samples was transferred directly to 4% formalin for 10 days, embedded in two blocks of paraffin (4–5 biopsies per block), and subsequently cut into 5 µm sections on a Microm HM340E (ThermoScientific) (**Figure 1** **C**).

### Immunostaining

Series of 5 µm adjacent sections were stained for GLP-2 immunoreactivity as a marker for L-cells. The current antibody has previously been shown to co-localize 100% with GLP-1 in brainstem preproglucagon expressing neurons [29]. In brief, sections were deparaffinized in toluene and rehydrated in series of ethanol. Sections were subjected to antigen retrieval in citrate buffer, blocked for endogenous peroxidase activity and non-specific binding before being incubated with primary mouse anti-GLP-2 antibody (1∶16000, GLP2-12F21-A7, kindly provided by Jes Thorn Clausen, Novo Nordisk A/S) for 1 hour. Sections were visualized using Envision (DAKO K4007) and finally developed using diaminobenzidine as a chromagen. Slides were counter-stained in hematoxylin before they were dehydrated and coverslipped with Pertex (Sakura, Denmark).

### Stereological Quantification of Volume

Stereological volume estimations were performed using newCAST software (Visiopharm, Copenhagen, Denmark) on digital slides scanned with a 20× objective on an Aperio Scanscope AT slidescanner (Aperio, California, USA). Images for counting were sampled in a random systematic way by use of the newCAST software. Total gut volume and cellular layer volumes were estimated by point counting using a grid system where all points hitting the structure of interest were counted. The volume of the different cell layers was estimated using a 16-point grid at approximately 650× magnification designed to ensure the most efficient precision [30]. The serosa layer was included as a part of the muscular layer. Tissue processing for paraffin embedding leads to substantial shrinkage in the tissue. In order to compensate for shrinkage we corrected the volume data by a shrinkage coefficient. This shrinkage coefficient was based on estimations of in-section shrinkage (i.e. in the XY axis) and length. The XY shrinkage induced by dehydration and paraffin embedding was estimated by comparing point counts performed on frozen unshrunken sections with paraffin embedded sections. The length shrinkage was estimated by subjecting 2 cm long agarose embedded segments to dehydration before re-measuring the length.

### Stereological Quantification of Cells

The total number of GLP-2 immunoreactive L-cells was estimated using the principle of the physical disector [31], [32], [33]. This method relies upon the principle that if a particle is seen in one section and not the previous, it is counted. For this purpose tissue sections were sampled as two consecutive sections, thus obtaining two overlapping adjacent sections on one slide. The slides were stained for GLP-2 immunoreactivity (-ir) as described above, scanned and digitized using a Aperio Scanscope AT slidescanner with a 20× objective and finally analyzed on a computer running CAST software package.

### qPCR

Frozen biopsies were placed in tubes with TRIzol reagent (Invitrogen 15596-026) and lysing matrix. Samples were homogenized using a FastPrep-24 and total RNA was purified according to manufactures specifications. RNA total content and quality was determined by absorbance (NanoDrop Technologies). cDNA was synthesized from 2 µg of RNA using SuperScript® II (Invitrogen 18064-022) reverse transcriptase according to manufacturers specifications. cDNA was diluted 30-fold. QPCR was performed using SYBR green (Stratagene 600828). Total reaction volume was 25 µl per reaction. 18s was used as an internal standard. The following primer sequences were used: PYY: TGCTCTTCACAGACGACAGC and CATGCAAGTGAAGTCGGTGT, preproglucagon: CTCTGGTGGCAAGGTTATCG and CATTCACAGGGCACATTCAC, 18S: TGTCAATCCTGTCCGTGTCC and ACGGACCAGAGCGAAAGCAT. Total expression levels were calculated as relative expression multiplied by total mucosa volume. The mean expression per cell was calculated by dividing total expression with total number of cells.

### Stereological Error and Statistics

The precision of the estimator was evaluated in comparison to the biological variability in a previous study. In this study the mean regional coefficient of error (CE) was calculated was found to be 0.07 [34]. All stereological estimates were based on 9–12 sections per region with a mean number of 428 counting events for the volumetric quantification, and 122 counting events per region for number estimation providing a coefficient or error below 0.10 [28]. The shape factor for the physical dissector was estimated to 25.

Data are presented as mean ± SEM. Statistical significance between groups was determined by one-way ANOVA followed by Tukey’s post-hoc analysis, or two-way ANOVA followed by Bonferroni post-hoc analysis. P-value below 0.05 was considered statistically significant.

## Results

### Body Weight and Food Intake

**Figure 2:**
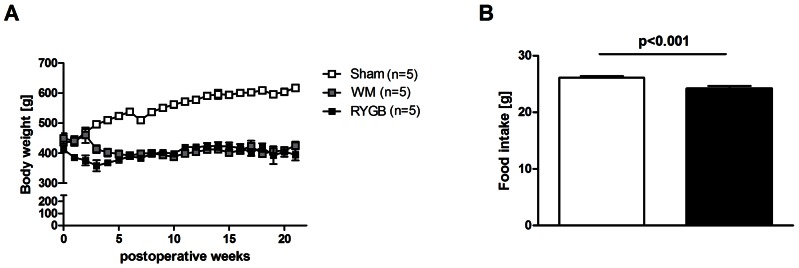
Body weight change for gastric bypass (n = 5) and sham-operated rats ad libitum fed (n = 5) and sham-operated body weight matched (n = 5) (A). Average daily food intake over 20 weeks for sham operated ad libitum fed rats and for gastric bypass rats (B).

After a short initial period of postoperative weight loss, SHAM rats returned to normal weight gain for the remainder of the study. At the day of termination, the body weight in the SHAM group was significantly higher than in the RYGB group (SHAM 617±10.8 and RYGB 395±19.2; p<0.001) (**Figure 2**). There was no significant difference in body weight between the RYGB and the SHAM WM on the day of termination (SHAM WM 426±12.6 g, p = 0.339) (**Figure 2**). The SHAM and RYGB group had an average spontaneous daily food intake of approximately 26 and 24 grams per day, respectively (p<0.001) (**Figure 2**), while the WM group received approx. 15 grams of chow per day.

### Gut Morphometry

**Figure 3:**
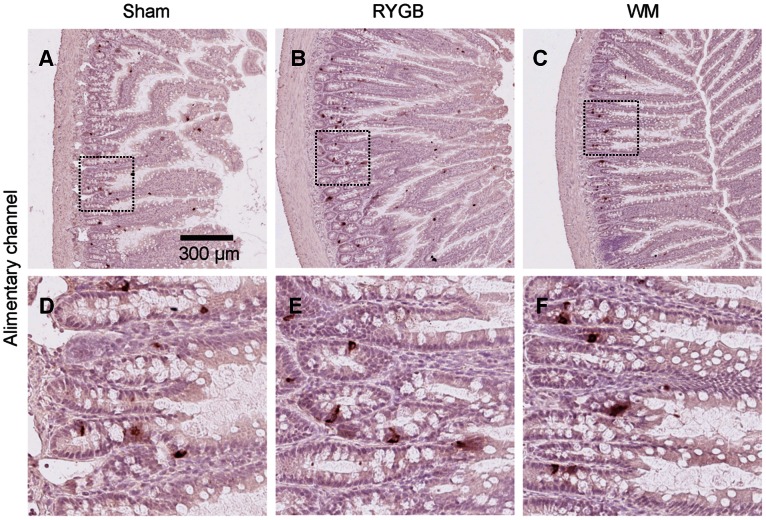
Representative micrographs of gut morphology (A–C) and GLP-2 immunohistochemistry (D–F) in the alimentary channel of SHAM, RYGB and SHAM WM animals.

**Figure 4:**
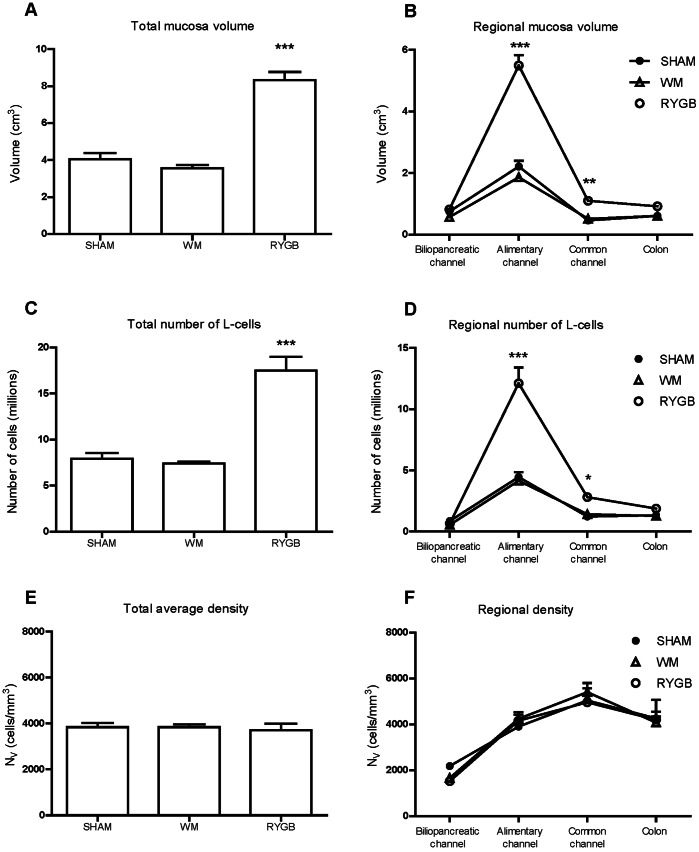
Stereological estimates of total and regional gut volume (A–B), L-cell number (C–D) and L-cell density (E–F). All values are presented as mean ± SEM. One-way ANOVA with Tukey's Multiple Comparison post-hoc test, (* = p<0.05,** = p<0.01, *** = p<0.001 for significance).

The length of the alimentary channel was increased by nearly 20% in the RYGB group compared to SHAM, and decreased by 10% in the SHAM WM group (**Table 1**). In contrast, the length of the biliopancreatic channel, common channel and colon appeared to be unaffected by surgery or food restriction (**Table 1**). A qualitative assessment of gut morphology in cross sections revealed a noticeable hypertrophy of the gut in RYGB operated rats compared to SHAM and SHAM WM groups. These changes were most noticeable in the alimentary channel as a thickening of the mucosa layer with elongated villi and deeper epithelial crypts (**Figure 3** **A–C**). The stereological assessment of total gut mucosa volume demonstrated an increase of more than 100% in the RYGB group compared to SHAM (p<0.001) (**Figure 4** **A**). The marked mucosal hypertrophy in RYGB animals was observed in all food exposed gut regions (alimentary channel, common channel and colon) but not the bypassed biliopancreatic limb (**Figure 4** **B**). The greatest increase in RYGB mucosa volume was observed in the alimentary channel (150%, p<0.001), followed by the common channel (140%, p<0.01) and colon, though the latter did not reach statistical significance (50%, p>0.05) (**Figure 4** **B**). In WM animals, the biliopancreatic and alimentary channel tended to have a slightly reduced mucosa volume compared to SHAM (**Figure 4** **B**).

**Table 1:** Length of gut segments.

	SHAM	WM	RYGB
Biliopancreatic channel	19.9±1.2	19.9±1.0	19.0±0.50
Alimentary channel	71.3±2.6	63.9±3.1*	85.6±4.8*
Common channel	21.3±1.1	23.1±0.9	21.4±0.42
Colon	17.8±0.73	16.9±0.72	19.4±0.65
Total	130.3±4.2	115.8±9.2	145.4±5.6

All values are presented as mean ± SEM. Significant differences were observed. Statistical analysis: One-way ANOVA with Tukey's Multiple Comparison post-hoc test, (* = p<0.05, for significance).

### Total and Regional Number of L-cells

L-cells were clearly identified by GLP-2 immunohistochemistry (**Figure 3** **D–E**). Similar to mucosal volume, the stereological quantification of L-cell numbers revealed a doubling in the total number of L-cells in the RYGB animals compared to SHAM (7.9±0.6 mill vs. 17.5±1.5 mill. p<0.001) (**Figure 4** **C**). The greatest increase in L-cell number was confined to the alimentary channel (170%, p<0.001) followed by the common channel (130%, p<0.05) and the colon, though the latter did not reach statistical significance (40%, p>0.05). No change was observed in the biliopancreatic limb. In general, the WM control group displayed no changes in total or regional L-cell number as compared to the sham group (**Figure 4** **D**).

Total and regional L-cell density remained unchanged among groups (**Figure 3** **D–F,** **Figure 4** **E**). In general, the L-cell density was lowest in the duodenal region with increasing density along the proximal-distal axis (**Figure 4** **F**). L-cell density was highest in the distal jejuno-ileum (common channel) and was slightly lower in the colon.

### Preproglucagon and PYY Gene Expression

**Figure 5:**
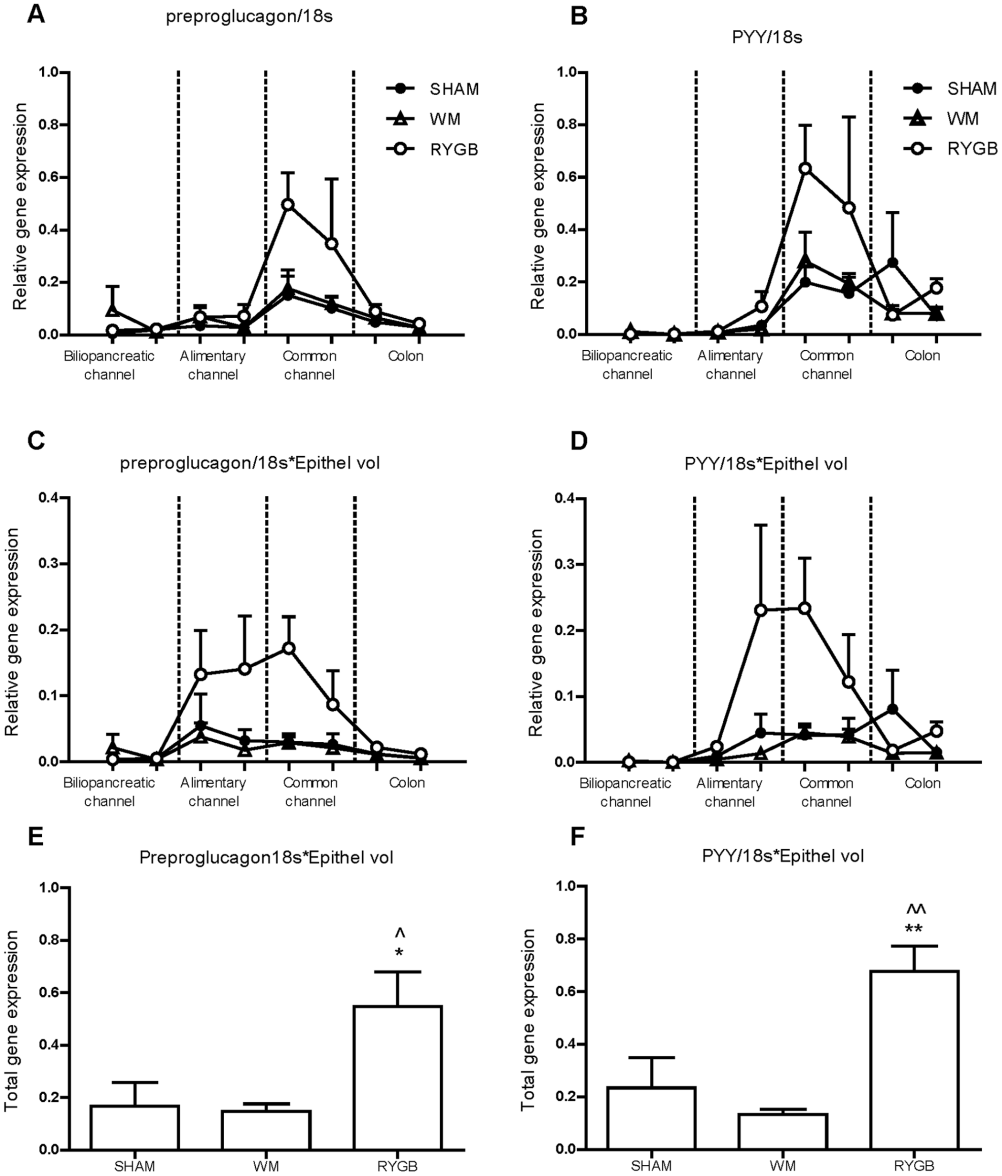
Regional preproglucagon (A) and PYY (B) gene expression normalized to 18s and corrected for the varying epithelial volume (C–D). Total preproglucagon (E) and PYY (F) gene expression. All values are arbitrary units presented as mean ± SEM. Statistical analysis: One-way ANOVA with Tukey's Multiple Comparison post-hoc test, SHAM vs. RYGB (*); VW vs. RYGB (?). (*? = p<0.05,**?? = p<0.01, for significance).

qPCR analysis of preproglucagon and peptide YY expression in the eight different gut segments was used to examine the relative mRNA levels along the rostro-caudal axis of the gut. This analysis revealed a nonsignificant tendency towards increased gene expression in the common channel at the level of confluence of food, gastric juices, bile and pancreatic enzymes (**Figure 5** **A–B**). When comparing the mean regional expression per cell (total preproglucagon expression/number of cells) gene expression in the common channel in the RYGB group was more than 4 times higher than the corresponding region in the SHAM animals (data not shown). In order to get an estimate of the total preproglucagon and PYY mRNA levels in the different gut regions, we incorporated regional volume changes into the calculation (**Figure 4** **C–D**). When mucosal volume was taken into consideration, this revealed a rise, which however was not significant, in total preproglucagon and PYY gene expression in both the alimentary channel and the common channel (**Figure 5** **C–D**). As seen in Figure 4E and 4F, total PYY (p<0.01) and preproglucagon (p<0.05) mRNA expression was significantly two-fold increased in the RYGB compared to the SHAM group (**Figure 5** **E–F**). In contrast, no changes were observed in the SHAM WM animals (p>0.05).

## Discussion

The present study provides the first stereological report of morphological changes in the rat gut after RYGB surgery. We show that this particular type of bariatric surgery results in a more than doubling of total mucosa volume in the small intestine with concomitant increases in the absolute number of L-cells, without affecting L-cell density. The observation that the alimentary region contains by far the highest number of L-cells in both the normal and gastric bypass operated gut is in contrast to the general view that L-cells are confined to the ileum and colon [35], [36]. Finally, we demonstrate that following RYGB both preproglucagon and PYY mRNAs are upregulated in the L-cells located in the common channel where food is mixed with bile acids, gastric and pancreatic secretions and enzymes.

A number of preclinical and clinical studies have indicated that bariatric surgery leads to structural changes in gut morphometry [20], [37], [38], [39], [40]. Using conventional quantitative methods, villus lengths, crypt depth and crypt proliferation have been reported to be increased in nutrient-stimulated regions in rat models of RYGB [22], [23], [37], [38], biliopancreatic diversion [20], [39] and ileal transposition [40]. Similarly, increased villus height and gut length have been reported in humans following jejuno-ileal bypass surgery [41], [42], [43]. The present rat study is in close agreement with the finding that RYGB in human individuals leads to substantial and regional selective hypertrophy of the gut. Notably, we demonstrate by the use of stereological methods, that RYGB leads to L-cell hyperplasia. The remarkable increase in the number of L-cells by a factor of about 2.5 (7.9±0.6 mill vs. 17.5±1.5 mill. p<0.001) underscores the marked plasticity of the gut in response to surgical interventions that alter nutrient flow. In contrast, no changes were observed in L-cell density. Therefore, it appears that L-cell densities are genetically encoded in the general mucosa turnover, and that L-cell hyperplasia is secondary adaptive mechanism to the marked changes in gut volume. A similar mechanism has also recently been suggested by Mumphrey and coworkers [22]. The increase in L-cell number reported in the present study does not account for the elevated postprandial GLP-1 and PYY plasma levels observed after RYGB in rodents and humans alone [44]. In this respect it should be noted that the mRNA transcriptional activity of the L-cells was increased in the common channel after RYGB hereby representing an additional potential mechanism leading to increased release of GLP-1 and PYY.

The reported increase in L-cell density along the rostro-caudal axis of the small intestine and the colon is in line with many previous reports [35], [36]. However, the present study present novel data on the absolute endocrine cell numbers in each region. These measurements incorporate the size and the length of each respective region and point to the alimentary channel as the main L-cell contributor after RYGB. Hence, total L-cell numbers in the alimentary limb are higher than the more distal gut regions despite the lower L-cell density. These data indicate that the main part of the L-cell population is actually found proximal to the traditionally defined ileum - in contrast to the generally held view [45] – but in line with other recent data from our laboratory [34]. However, it should be recognized that the distribution between RYGB regions may vary depending on the surgically defined length of each gut segment.

The nature of the signals leading to intestinal hypertrophy remains to be identified. It is tempting to speculate that mechanical or nutrient induced stimuli caused by the introduction of undigested food particles in the upper jejunum are involved; this would also explain the lack of hypertrophy in the nutrient diverted biliopancreatic limb observed in this study and by others [22]. Increased villus length and increased cell number have been demonstrated in mice fed a high-fat diet for 8 weeks [46]. Moreover, hypertrophy has also been described from a number of small bowel resection studies [47], [48] where the remaining small intestine increases in wall thickness, villus height and total weight. This adaptation compensates to some extent for the lost nutrient absorption capacity in the resected small bowel.

It is remarkable that hormonal changes are triggered with simultaneous improvement in glucose homeostasis prior to significant weight loss following RYGB [49]. Increased GLP-1 secretion is considered the primary contributor in mediating the rapid improvements in glycemic control [50], [51]. Hence, to elucidate a possible mechanism linked to the highly elevated levels of circulating GLP-1 and PYY in our RYGB rat model [11], [52], we investigated not only changes in the absolute number of L-cells following RYGB but also preproglucagon and PYY expression in each intestinal segment. Whereas the alimentary and common channel contributed equally to changes in total preproglucagon and PYY expression, increased mRNA expression per cell was only seen in the common channel where the biliopancreatic channel is fused with the alimentary channel. In this region, macronutrients are mixed with bile acids, gastric and pancreatic juices, suggesting that these digestive components directly stimulate L-cell gene expression in this region. Accordingly, bile acids have previously been shown to increase L-cell secretion [53], [54], [55] through the bile specific G-protein coupled receptor TGR5 [53]. This receptor is involved in a range of beneficial metabolic effects including weight gain resistance, and maintenance of glucose homeostasis and insulin sensitivity [56].

A secondary effect of increased L-cell stimulation is secretion of GLP-2. GLP-2, a prominent mediator of epithelial proliferation [57] synthesized and secreted from the L-cell in a 1∶1 stoichiometric ratio with GLP-1 [58], [59], may also play a central role in the mucosal hypertrophy. In this respect it should be noted that elevated GLP-2 plasma measurements previously has been documented in this specific RYGB model [26]. Another possible mechanism stimulating both intestinal hypertrophy and L-cell secretion are changes in gut microbiota [60]. More studies are however needed to evaluate the exact mechanism leading to changes in gut microbiota following RYGB surgery, and to elucidate the direct or indirect effects of these changes on the endocrine cells.

All endocrine cells are believed to originate from the same precursor cells [61]. Consequently, it is plausible that other gut enteroendocrine, e.g. GIP-, neurotensin and CCK cells, are affected by the gut hypertrophy following RYGB. As most of the endocrine cell types in the intestine produce hormones with anorectic effects, and many hormones are elevated in postprandial plasma after surgery, gut hypertrophy may have important implications for unraveling the anti-diabetes/anti-obesity effect of RYGB surgery [62], [63]. Additional studies focusing on other endocrine markers are warranted to substantiate this hypothesis.

Our findings in this report are subject to some limitations. First, our data apply to rats and the effects observed on intestinal hypertrophy and L-cell numbers seen in this study have not yet been reported in humans. However, comparable changes in plasma hormone levels in humans and rats after RYGB imply that similar changes in the human endocrine cell populations may also occur. Secondly, the rats used were not obese or on a high-fat diet. Even though sham animals gained more than 200 grams over the other groups and were significantly heavier than the other groups at the time of study termination, we cannot exclude that our findings would have differed slightly had our rats been fed a high fat diet. Thirdly, the current study did not examine hormonal plasma levels. Although these have previously been measured and although changes after RYGB are very consistent across published studies, we cannot make a direct link between our findings of increased L-cell numbers in the gut and plasma concentrations of the respective hormones in the present study. Hence, we cannot directly correlate the L-cell mass or mRNA transcripts to circulating PYY and GLP-1 levels. Finally, it should be noted that we used non-diabetic chow fed rats in our study. Hence, the effect observed on L-cell expression and L-cell hyperplasia cannot be directly coupled to glycemic improvements in obese T2DM patients undergoing RYGB.

In conclusion, RYGB in rats is associated with gut hypertrophy, an increase in L-cell number and increased preproglucagon and PYY gene expression. The data suggest that elevated GLP-1 and PYY hormone levels after RYGB surgery are a consequence of L-cell proliferation secondary to gut hypertrophy and hereby provide further insight into the possible mechanisms underlying the marked remission of T2DM following RYGB.
